# Supplementary material for: Mitigation of China’s carbon neutrality to global warming
Source: Nat Commun. 2022 Sep 9;13:5315. doi: 10.1038/s41467-022-33047-9 (PMC9463144; doi:10.1038/s41467-022-33047-9)
Supplement: Supplementary file 1 — Supplementary Information [file 41467_2022_33047_MOESM1_ESM.pdf]

Supplementary for "Mitigation of China's carbon neutrality to global warming"

Longhui Li[1, 2, 3], Yue Zhang[1, 2, 3], Tianjun Zhou[4], Kaicun Wang[5], Can Wang[6], Tao Wang[7], Linwang Yuan[1, 2, 3], Kangxin An[6], Chenghu Zhou[8], Guonian Lü[1, 2, 3, *]

[1] Jiangsu Center for Collaborative Innovation in Geographical Information Resource Development and Application, Nanjing 210023, China

[2] Key Laboratory of Virtual Geographic Environment (Nanjing Normal University), Ministry of Education, Nanjing 210023, China

[3] School of Geographical Sciences, Nanjing Normal University, Nanjing 210023, China

[4] LASG, Institute of Atmospheric Physics, Chinese Academy of Sciences, Beijing, China

[5] Sino-French Institute for Earth System Science, College of Urban and Environmental Sciences, Peking University, Beijing, 100871, China

[6] School of Environment, Tsinghua University, Beijing, China

[7] State Key Laboratory of Tibetan Plateau Earth System and Resources Environment (TPESRE), Institute of Tibetan Plateau Research, Chinese Academy of Sciences, Beijing, China

[8] Institute of Geographical Information Science and Natural Resources, Chinese Academy of Science, Beijing, China

*Corresponding author: Guonian Lü, Email: gnlu@njnu.edu.cn

This supplementary includes:

Material and Methods

Table S1

Figures S1-9

Materials and Methods

Model simulations

To examine the individual contribution of China's Carbon Neutrality (CNCN) to global warming mitigations, 4 pairs of simulations based on CESM 2.1.3 are implemented. Each pair of simulations consist in a default CESM simulation and a CNCN one. In the default simulations, anthropogenic surface CO_2 emission data is from the default shared socioeconomic pathway (SSP). In this study, 4 SSPs, i.e. SSP1-2.6, SSP2-4.5, SSP3-7.0 and SSP5-8.5, are used. In the CNCN simulations, the anthropogenic surface CO_2 emission is replaced by the values in the scenario of CNCN. The 4 pairs of CESM simulations are run at spatial resolution of 1.9°×2.5° (Table S1).

Table S1. Eight CESM simulations used in this study. The CMIP6 simulation is referred to as the default shared socioeconomic pathway (SSP) experiment for the Coupled Model Intercomparison Project Phase 6 (CMIP6) based on the CESM2.1.3, and the CNCN simulation is referred to as the CMIP6 simulation with replacement of anthropogenic CO_2 emissions under the China's Carbon Neutrality (CNCN) scenario.

No.	Simulations	Spatial Res.	Explanation
**1**	SSP1-2.6 CMIP6	1.9°×2.5°	The default SSP1-2.6 experiment for CMIP6
**2**	SSP1-2.6 CNCN	1.9°×2.5°	SSP1-2.6 CMIP6 with anthropogenic CO_2 emissions under China's Carbon Neutrality (CNCN) scenario
**3**	SSP2-4.5 CMIP6	1.9°×2.5°	The default SSP2-4.5 experiment for CMIP6
**4**	SSP2-4.5 CNCN	1.9°×2.5°	SSP2-4.5 CMIP6 with anthropogenic CO_2 emissions under China's Carbon Neutrality (CNCN) scenario
**5**	SSP3-7.0 CMIP6	1.9°×2.5°	The default SSP3-7.0 experiment for CMIP6
**6**	SSP3-7.0 CNCN	1.9°×2.5°	SSP3-7.0 CMIP6 with anthropogenic CO_2 emissions under China's Carbon Neutrality (CNCN) scenario
**7**	SSP5-8.5 CMIP6	1.9°×2.5°	The default SSP5-8.5 experiment for CMIP6
**8**	SSP5-8.5 CNCN	1.9°×2.5°	SSP5-8.5 CMIP6 with anthropogenic CO_2 emissions under China's Carbon Neutrality (CNCN) scenario

Anthropogenic surface CO_2 emissions under CNCN scenario

In October 2020, a research team in Tsinghua University released a synthesis report, Strategies and Transmission Pathway for China's Long-term Low Carbon Development (Ref S1). And this report was later published in a Chinese journal, China Population, Resources and Environment in November 2020 (Ref S2). In this report, a first roadmap on CNCN, i.e. anthropogenic CO_2 emission data from 2015 to 2050 was projected.

The CNCN scenario is mainly based on carbon emissions consistent with the IPCC 1.5 °C target, but it requires further reductions in national total energy consumptions and large increases in the proportion of non-fossil energy to primary energy consumptions. The CNCN scenario also requires significant decreases in non-CO_2 GHG emissions and increases in terrestrial ecosystem carbon sinks, and large-scale implementations of carbon capture and storage (CCS) and carbon dioxide

removal (CDR). Anthropogenic surface CO_2 emission data under the CNCN scenario is a total value of the entire China ($CO_{2,CNN}$), so it has to be converted into spatial domain in China. To do this, we assume that future anthropogenic CO_2 emission for each grid under CNCN ($CO_2(i,j)$) is linearly proportional to its original SSP values ($CO_{2,SSP}(i,j)$), i.e. the Equation S1 below.

$$CO_2(i,j) = \frac{CO_{2,CNN}}{\sum_1^n CO_{2,SSP}(i,j)} \times CO_{2,SSP}(i,j) \quad \text{(S1)}$$

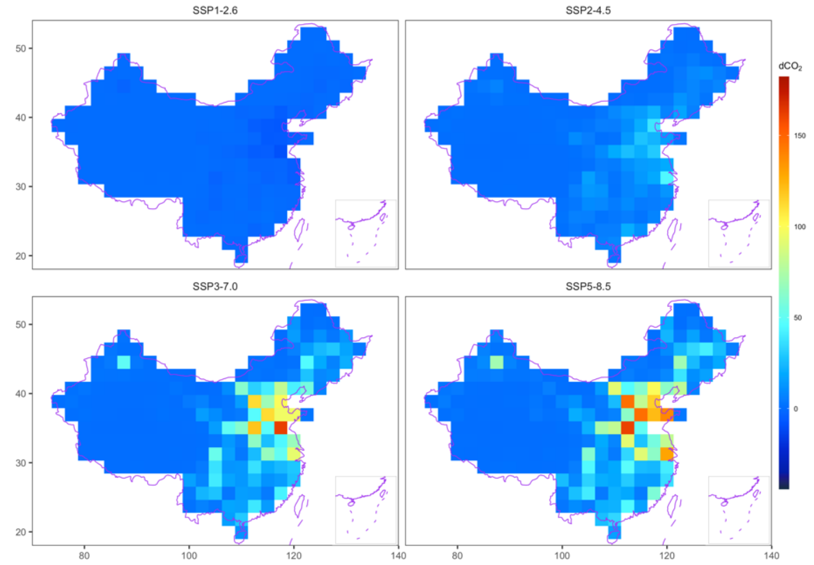

Figure S1. Difference of China's anthropogenic CO_2 emissions between the default CMIP6 and CNCN scenarios (dCO_2) for 4 SSPs in the year 2070. The unit of dCO_2 is $kgCO_2\ m^{-2}\ year^{-1}$.

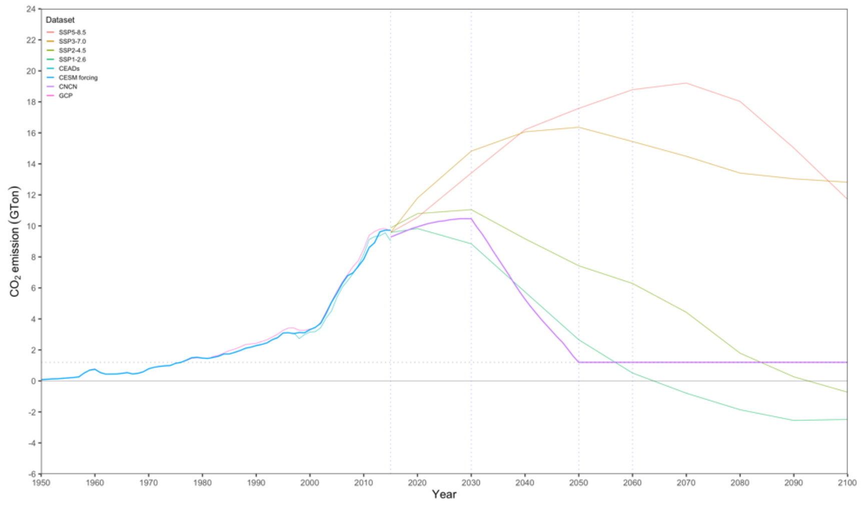

Figure S2. China's anthropogenic CO_2 emissions from 1950 to 2100. Before 2015, three independent datasets are used. The three datasets consist of the default CO_2 forcing data of CESM (CESM forcing), Global Carbon Project (GCP, http://www.globalcarbonatlas.org/en/CO2-emissions, Ref S3) and Carbon Emission Accounts & Datasets (CEADs, https://www.ceads.net/, Ref S4). After 2015, anthropogenic CO_2 emissions data from four SSPs and CNCN (Ref S1-2) are used.

Based on 4 SSP anthropogenic surface CO_2 emission data, we regenerated the anthropogenic surface CO_2 emission data from 2015 to 2100 in China's domain and replaced the China's CO_2 emission data but kept unchanged in other regions of the world. The new datasets are used to drive the CESM model for all 4 SSP scenarios and represent the CNCN simulations. Figure S1 illustrates the CO_2 emission difference (dCO_2) between the default SSP and CNCN scenarios for 2070 in China.

Figure S2 shows the China's anthropogenic CO_2 emissions from 1950 to 2100. Before 2000, China's anthropogenic CO_2 emission has increased steadily. After 2000, three datasets show consistent and large increase of anthropogenic CO_2 emissions, reaching about 10 $GtCO_2$ $year^{-1}$ (Gigatons). From present to the end of this century, China's anthropogenic CO_2 emission ranges from -2.55 $GtCO_2$ $year^{-1}$ for SSP126 in 2090 to 19.21 $GtCO_2$ $year^{-1}$ for SSP5-8.5 in 2070. Compared with CNCN scenario, China's carbon neutrality pledge could reduce emissions up to 18 $GtCO_2$ $year^{-1}$ (Figure S2).

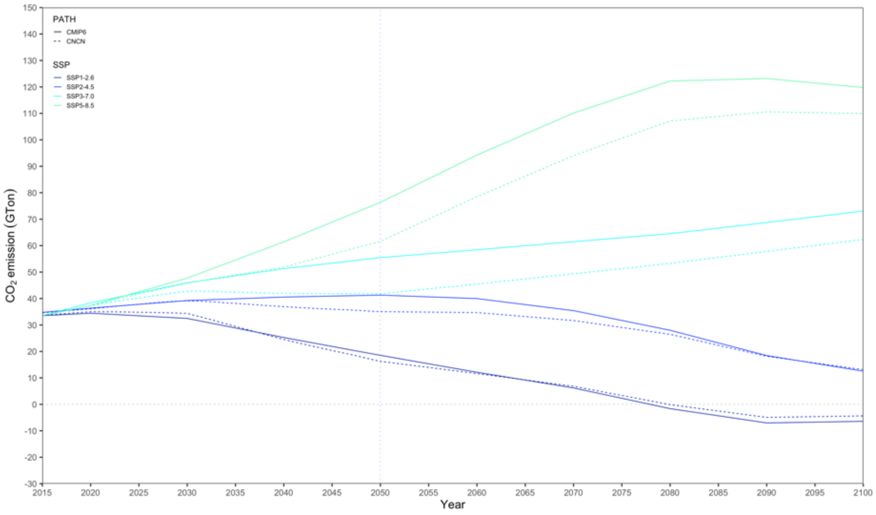

Figure S3. Total global anthropogenic CO_2 emission from 2015 to 2100 for four SSPs under the default CMIP6 and CNCN scenarios.

Contribution of CNCN to global anthropogenic surface CO_2 emissions varies for SSP and with year (Figure S3). Under two low forcing pathways (SSP1-2.6 and SSP2-4.5), the CNCN scenario differs not too large from the default CMIP6. But under two high forcing pathways (SSP3-7.0 and SSP5-8.5), CNCN could cause a reduction of global anthropogenic surface CO_2 emissions 19.21 $GtCO_2$ $year^{-1}$ for SSP5-8.5 in 2070. This amount of reduction in anthropogenic surface CO_2 emissions accounts for around 56% of currently global CO_2 emissions. It is expected that such a huge reduction in emissions of CO_2 may mitigate future global warming.

CH_4 and N_2O emissions and conversion from emissions to concentrations under CNCN scenario

Under the default CMIP6 scenarios, global annual CH_4 and N_2O emissions are shown in left panel of Figure S4. We can see that global annual CH_4 and N_2O emissions evolves largely different among four SSPs. Estimates of future CH_4 and N_2O emissions for different SSPs are derived from various global Integrated Assessment Models (IAMs) and these database are archived in IIASA

(https://tntcat.iiasa.ac.at/SspDb). Among those IAMs, China is generally considered as one or a quite similar geographical region, so that it is easy to extract their future variations of both CH_4 and N_2O from 2014-2100 in China's domain (right panel of Figure S4). As China doesn't specify non-CO_2 GHG (CH_4 and N_2O) emissions reduction target and timeline for GHG neutrality yet, an assumption of equative marginal cost at emissions reductions between CO_2 and non-CO_2 GHG is applied to run global change assessment model (GCAM, version 5.4) (Ref S5) and CO_2 emissions under the CNCN scenario is used as a constraint.

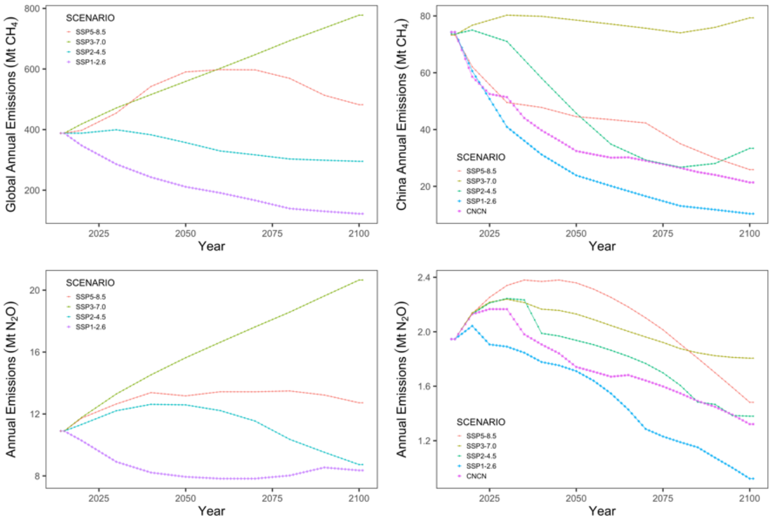

Figure S4. Annual emissions of CH_4 and N_2O for the four SSPs over the global and the four SSPs and CNCN scenarios in China from 2015 to 2100.

The first step of the procedure is to calculate out the carbon price based on the CNCN pathway. Following the equative marginal cost at emissions reductions between CO_2 and non-CO_2 GHG, the second step is to derive the prices of CH_4 and N_2O according to their corresponding global warming potentials (GWP). And finally, the GCAM is used to derive the emissions pathways of CH_4 and N_2O for the CNCN scenario (right panel of Figure S4).

Both global annual CH_4 and N_2O emissions for SSP3-7.0 roughly linearly increase with year, and those for other SSPs evolve differently each other. For the two primary non-GHGs, China's proportions account for about 10% of global share for each default SSP. Under the CNCN scenario, evolution curve of annual CH_4 emissions is ranked between SSP1-2.6 and SSP2-4.5 or SSP3-7.0, and N_2O evolves between SSP1-2.6 and SSP2-4.5.

Both CH_4 and N_2O forcing data of the CESM are prescribed as globally uniform surface concentrations, rather than their surface fluxes. Conversion from flux to concentrations generally requires to run a IAM like MAGICC (Ref S6). Running IAM has often required a large amount of simulation tasks. Because both CH_4 and N_2O concentrations in the troposphere have strong dependences (R^2 close to 1) on cumulative emissions (Figure S5) for four SSPs under the default CMIP6 scenario although their fitting equations are largely different among variables and SSPs, it is feasible to derive corresponding surface concentrations based on their cumulative emissions during a specified period (2015-2100) (Figure S5).

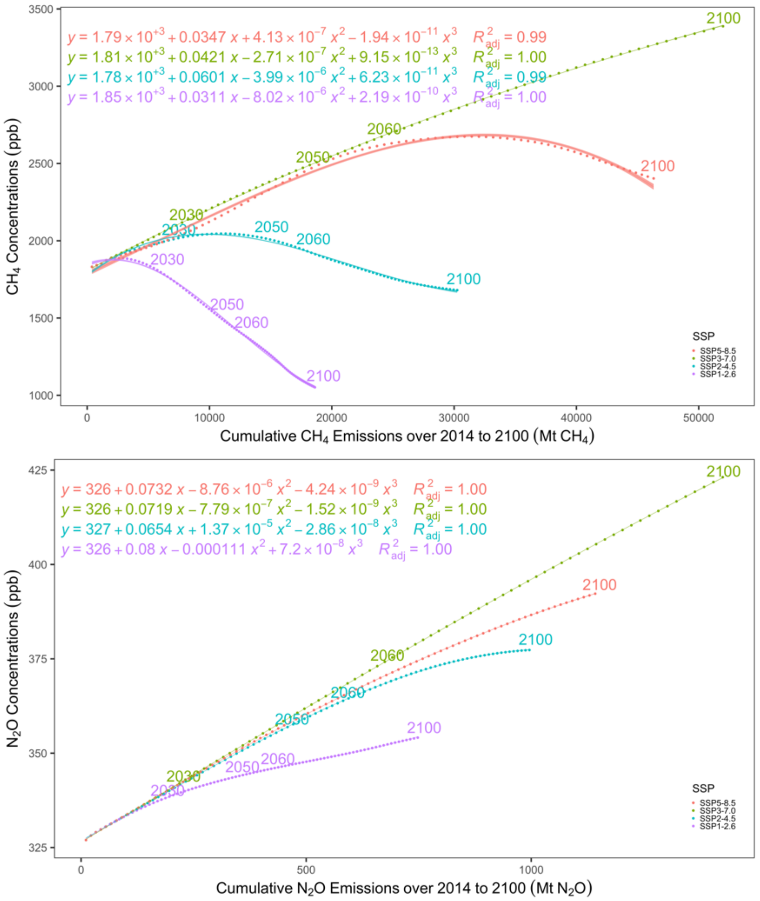

Figure S5. Dependence of global CH_4 (top) or N_2O (bottom) concentrations on cumulative emissions for the four SSPs under the default CMIP6 scenarios during the period 2015-2100.

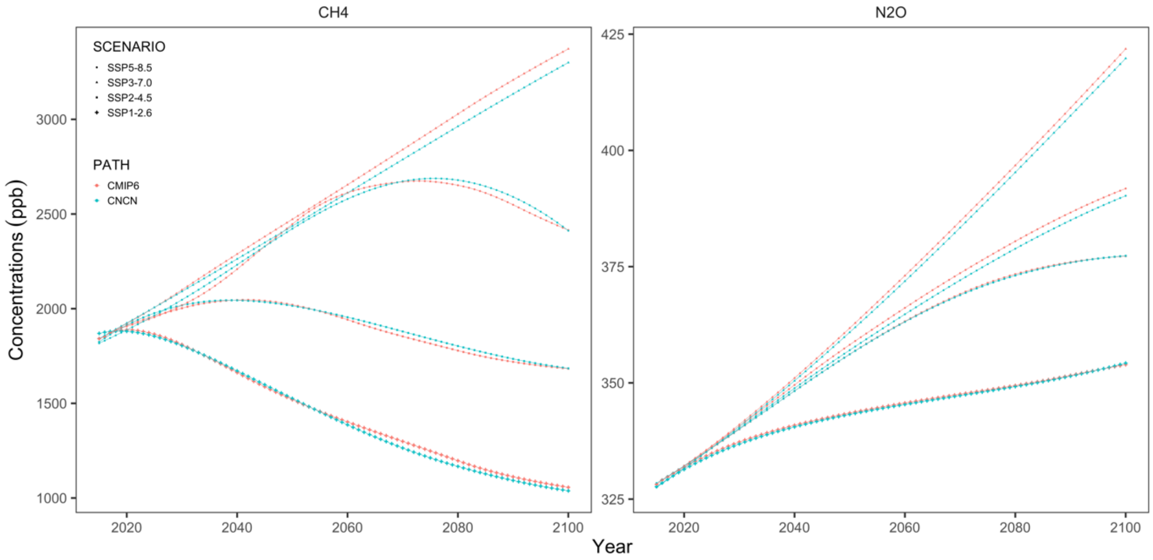

Figure S6. Comparisons of global CH_4 (left) and N_2O (right) concentrations between the default SSPs and corresponding CNCN scenarios during the period 2015-2100.

Once the fitting equations between surface concentrations and cumulative emissions under different SSPs are derived, surface concentrations of both CH_4 and N_2O can be calculated out based on their emissions under the CNCN scenario (Figure S6). As a result, CNCN doesn't actually results in large impacts on global CH_4 and N_2O concentrations for both SSP1-2.6 and SSP2-4.5 but caused remarkable differences in global CH_4 concentration (maximal 74 ppb for SSP3-7.0 and 41 ppb for SSP5-8.5) and N_2O concentrations (maximal 2.1 ppb for SSP3-7.0 and 1.7 ppb for SSP5-8.5) (Figure S6).

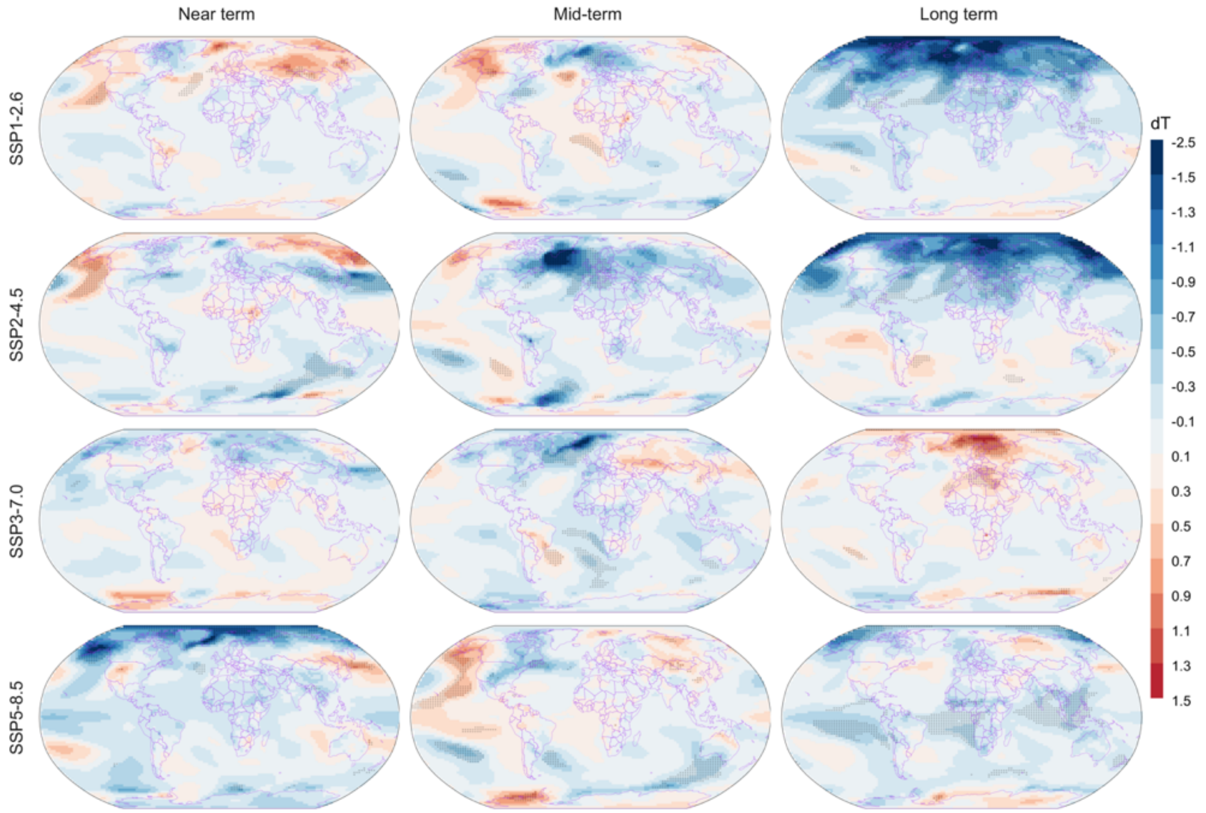

Figure S7. Mean surface temperature difference between the China's carbon neutrality (CNCN) and its extension scenarios with additional CH_4 and NO_2 emission reductions accompanied by the CO_2 emission reductions for China's carbon neutrality ($CNCN_{ext}$) for four SSPs. Panels from left to right represent near term (2021-2040), mid-term (2041-2060) and long term (2081-2100), and panel from top to bottom represent SSP1-2.6, SSP2-4.5, SSP3-7.0 and SSP5-8.5, respectively. Mean surface temperature difference (dT, unit in °C) is calculated as the value of $CNCN_{ext}$ minus CNCN for each combinations of study term and SSP. Pixels overlayed by sign of dot indicate that dT is statistically significant at confidence of 0.01. The paired t-test was used to calculate the significance.

Robustness of CESM

We run the CESM2.1.3 (Ref S7) with a full coupled components from 1850 and 1900 to 2014, and compare the global mean temperature (reference height temperature) with three historical global temperature datasets, including the Berkeley (http://berkeleyearth.org/data/), NASA GISTEMP

(https://data.giss.nasa.gov/gistemp/) and HadCRUT5.0 (https://crudata.uea.ac.uk/cru/data/temperature/#datdow) datasets (Figure S4). Generally, global mean temperature (including land and ocean) derived from the CESM agrees well with the three temperature datasets (Figure S4), particularly during the period 1930-2014 (linear correlation coefficient from 0.78 to 0.85) (Figure S5). These results justify the robustness of CESM in simulating long-term temperature.

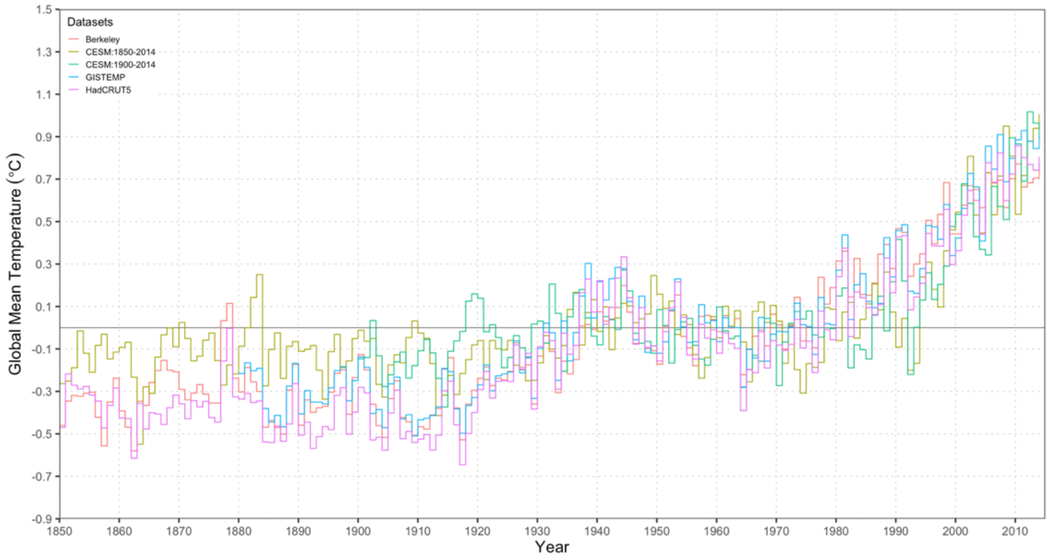

Figure S8. Anomaly of global mean surface temperature ("Global Mean Temperature" in the y-axis) simulated by CESM 2.1.3 and its comparison with three observation-based global temperature datasets, including the datasets from the Berkeley Earth, GISTEMP and HadCRUT5. The CESM model are run two times with starting year from 1850 (CESM:1850-2014) and 1900 (CESM:1900-2014) to 2014.

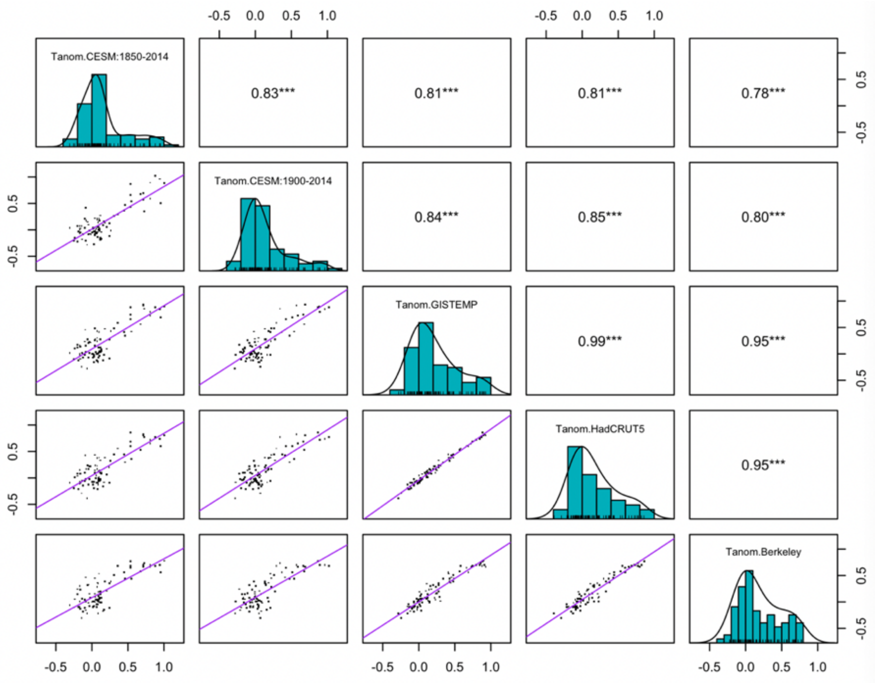

Figure S9. Scatter plot of relationship between global mean surface temperature anomaly simulated by CESM during the period 1930-2014 and three global temperature datasets. Shown in the diagonal panels is the histogram of temperature anomaly for each dataset. The number with sign of "***" shown in the upright panel represent the correlation coefficient and the corresponding *p* significance, and "***" denotes the *p* value less than 0.001.

References

S1. He JK. Introduction to Project Achievements: Research on China's Low-Carbon Development Strategy and Transformation Path. 2020; 15.

S2. Institute of Climate Change and Sustainable Development, Tsinghua University. Synthesis Report on Strategies and Transformation Pathway for China's Long-term Low Carbon Development. China Population, Resources and Development. 2021; **30**: 1-25.

S3. Friedlingstein P, O'Sullivan M, Jones MW, Andrew RM, Hauck J, Olsen A, Peters GP, Peters W, Pongratz J, Sitch S, Le Quéré C, Canadell JG, Ciais P, Jackson RB, Alin S, Aragão LEOC, Arneth A, Arora V, Bates NR, Becker M, Benoit-Cattin A, Bittig HC, Bopp L, Bultan S, Chandra N, Chevallier F, Chini LP, Evans W, Florentie L, Forster PM, Gasser T, Gehlen M, Gilfillan D, Gkritzalis T, Gregor L, Gruber N, Harris I, Hartung K, Haverd V, Houghton RA, Ilyina, T, Jain AK, Joetzjer E, Kadono K, Kato E, Kitidis V, Korsbakken JI, Landschützer P, Lefèvre N, Lenton A, Lienert S, Liu Z, Lombardozzi D, Marland G, Metzl N, Munro DR, Nabel JEMS, Nakaoka S-I, Niwa Y, O'Brien K, Ono T, Palmer PI, Pierrot D, Poulter B, Resplandy L, Robertson E, Rödenbeck C, Schwinger J, Séférian R, Skjelvan I, Smith AJP, Sutton AJ, Tanhua T, Tans PP, Tian H, Tilbrook B, van der Werf G, Vuichard N, Walker AP, Wanninkhof R, Watson AJ, Willis D, Wiltshire AJ, Yuan W, Yue X, and Zaehle S.Global Carbon Budget 2020. Earth Syst. Sci. Data. 2021; **12**: 3269–3340. https://doi.org/10.5194/essd-12-3269-2020.

S4. Chen J, Gao M, Cheng S, Hou W, Song M, Liu X, Liu Y, Shan Y. County-level CO_2 emissions and sequestration in China during 1997–2017. Sci Data.2020; **7**: 391 https://doi.org/ 10.1038/s41597-020-00736-3.

S5. Calvin K, Patel P, Clarke L, Asrar G, Bond-Lamberty B, Cui RY, *et al.* GCAM v5.1: representing the linkages between energy, water, land, climate, and economic systems. *Geoscientific Model Development* 2019, **12**(2)**:** 677-698.

S6. Meinshausen M, Nicholls ZRJ, Lewis J, Gidden MJ, Vogel E, Freund M, *et al.* The shared socio-economic pathway (SSP) greenhouse gas concentrations and their extensions to 2500. *Geoscientific Model Development* 2020, **13**(8)**:** 3571-3605.

S7. Danabasoglu G, Lamarque JF, Bacmeister J, Bailey DA, DuVivier AK, Edwards J, *et al.* The Community Earth System Model Version 2 (CESM2). *Journal of Advances in Modeling Earth Systems* 2020, **12**(2).
